# A Novel Colorimetric Fluorescent Probe for SO_2_ and Its Application in Living Cells Imaging

**DOI:** 10.3390/molecules23040871

**Published:** 2018-04-10

**Authors:** Ming-Yu Wu, Jing Wu, Yue Wang, Yan-Hong Liu, Xiao-Qi Yu

**Affiliations:** 1 School of Life Science and Engineering, Southwest Jiaotong University, Chengdu 610031, China; w1249280681@sina.com (J.W.); chinakywy2009@163.com (Y.W.); 2 Key Laboratory of Green Chemistry and Technology, Ministry of Education, College of Chemistry, Sichuan University, Chengdu 610064, China; yanhongliu@scu.edu.cn

**Keywords:** fluorescent probe, SO_2_, colorimetric, living cell imaging

## Abstract

A novel chromenylium-based fluorescent probe was exploited for sulphur dioxide (SO_2_) detecting. The probe displayed a remarkable fluorescence turn-on response towards SO_2_ based on the nucleophilic addition reaction to the carbon-carbon double bond with 105 nm Stock shift. The probe was successfully applied for the quantification of SO_2_.The linear detection range was from 0–160 μM with the detection limit as low as 99.27 nM. It also exhibited high selectivity for SO_2_ than other reactive species and amino acids. Furthermore, cell staining experiments indicated that the probe was cell membrane permeable and could be used for high-performance imaging of SO_2_ in living cells. The superior properties of the probe made it highly promising for use in chemical and biological applications.

## 1. Introduction

As one of well-known air pollutants, sulphur dioxide (SO_2_) has been studied extensively in toxicology [1]. However, recent works show that SO_2_ is generated endogenously and mainly from sulphur-containing amino acids through biosynthetic pathways, such as transamination by aspartate aminotransferase (AAT) [2,3]. Moreover, SO_2_ play significant roles in physiological processes especially in the regulation of cardiovascular function in synergy with NO and the lowering of blood pressure [4,5]. The abnormal endogenous levels of SO_2_ are believed to be linked with lung cancer, cardiovascular disease, and a number of neurological disorders [6,7,8,9,10]. Toxicological studies further suggested that SO_2_ and its derivatives could change the characteristics of voltage-gated sodium channels and potassium channels in rat hippocampal neurons [11], affect thiol levels and, hence, affect redox balance in cells [12] and produce a neuronal insult [13]. These observations have led to speculation that SO_2_ in the fourth gasotransmitter [14]. Thus, it is of great importance to develop novel analytical methods for the study of the biological and pathological roles of SO_2_ in physiological systems [15,16].

In recent years, fluorescence probes attract a great deal of attention due to the superiorities of simple operation, good selectivity, and high sensitivity as well as noninvasive imaging of biological molecules and processes with high spatial and temporal resolution in real-time [17,18,19,20]. For SO_2_ detection, it was mainly based on the nucleophilic addition reaction to the aldehydes, ketones, or carbon-carbon double bonds [21,22,23,24,25,26,27,28,29,30,31,32,33,34,35,36,37]. Some of them had been applied for detection of SO_2_ in organelles or in tumors [24,25,26,36]. However, some drawbacks of those probes are difficult to avoid, such as the small Stokes shift, high detection limit, poor selectivity, remarkable interference from biothiols, or reactive oxygen species, and so on. Therefore, it is necessary to exploit more efficient fluorescent probes for SO_2_ and its derivatives with larger Stokes shifts and stronger intensities, in particular, with high sensitivity and selectivity.

**Scheme 1:**
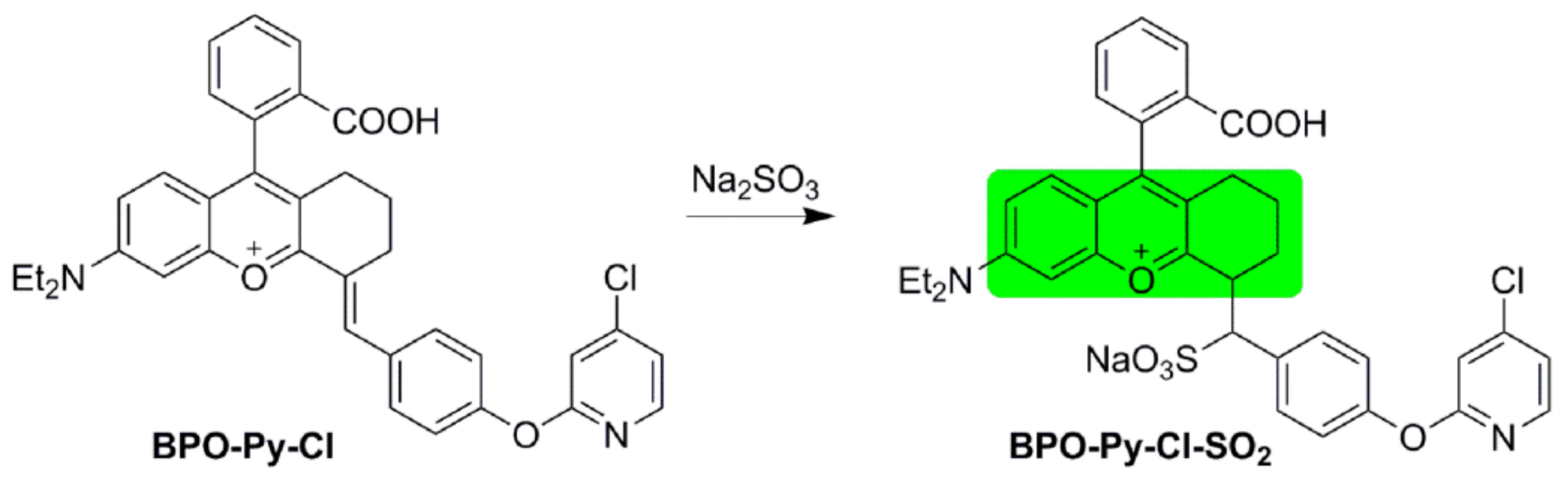
The detection process of **BPO-Py-Cl** with SO_2_.

In recently years, Yuan and Lin developed a series of chromenylium-based fluorescent probes with larger Stokes shifts, substantial quantum yields, and photostability under physiological conditions which are widely used to detection and imaging of bio-active species in vivo, such as H_2_O_2_, HClO, biothiols, H_2_S, Sec, and so on [38,39,40,41]. Intrigued by their findings, we designed one novel fluorescent probe for SO_2_ by modifying chromenylium with an electron-withdrawing group (substituted pyridine) to synthesize **BPO-Py-Cl**, which could be a selective probe to detect SO_2_ in HeLa cells based on the nucleophilic addition reaction to the carbon-carbon double bond (Scheme 1).

## 2. Results

### 2.1. Synthesis and Characterization of ***BPO-Py-Cl***

**Scheme 2:**
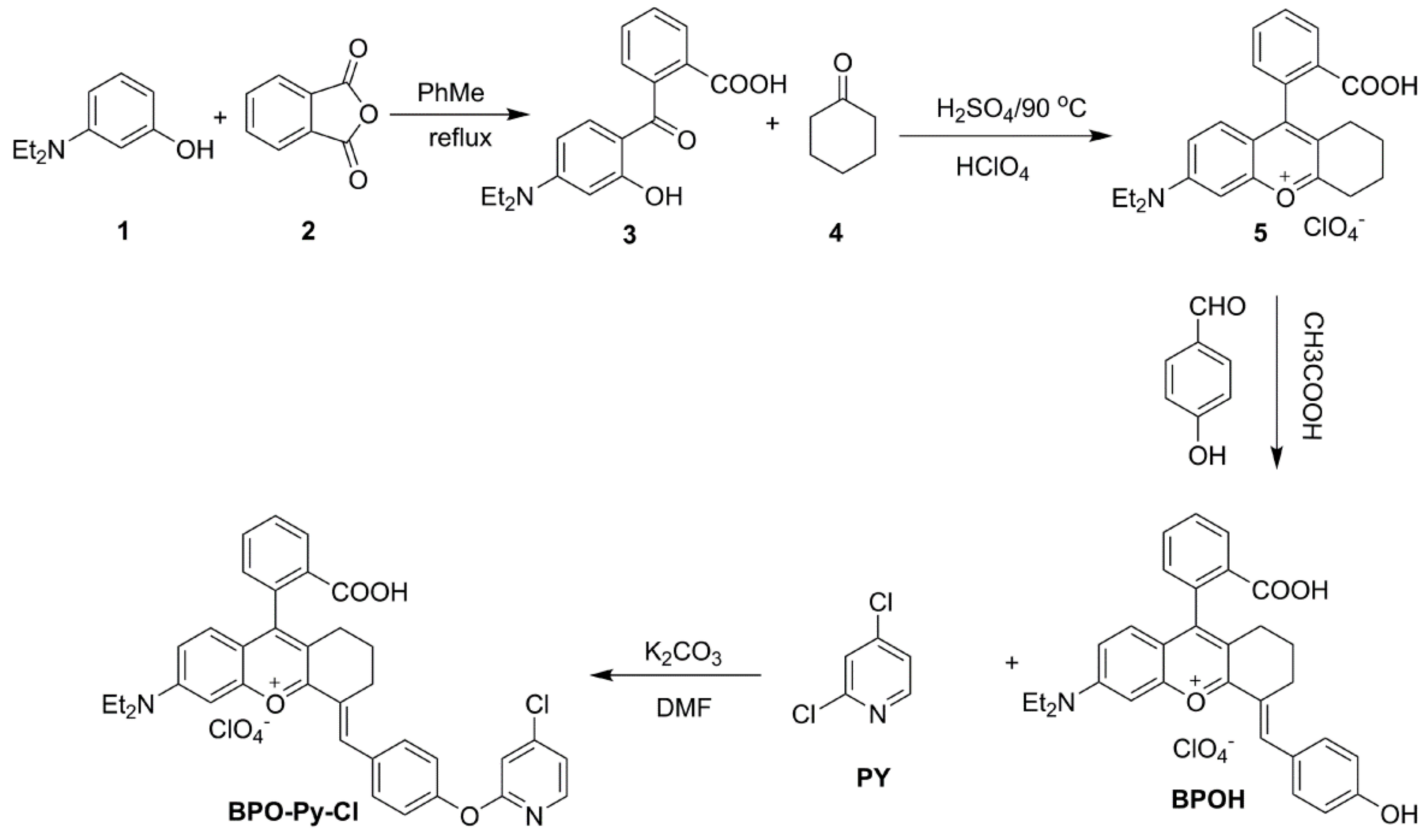
Synthesis route of fluorescent probe **BPO-Py-Cl**.

The synthesis route of the target molecule is illustrated in Scheme 2. It takes four steps to complete its composition, as follows: **BPOH** was synthesized according to the literature [40,42]. First, the Friedel-Crafts acylation reaction of 3-(diethylamino)phenol with phthalic anhydride can obtain compound **3** which could further react with cyclohexanone by a two-step cascade reaction to acquire oxonium **5**. Then, a condensation reaction between 4-hydroxybenzaldehyde with compound **5** will produce **BPOH**. Finally, **BPO-Py-Cl** could be obtained by a nucleophilic substitution reaction of **BPOH** with the 2-chloropyridine derivative. The probe has been characterized by ^1^H-NMR, ^13^C-NMR, and HRMS. Detailed synthetic procedures and structure characterizations were given in the experimental section and supporting information.

### 2.2. Optical Response of ***BPO-Py-Cl*** toward SO_2_

**Figure 1:**
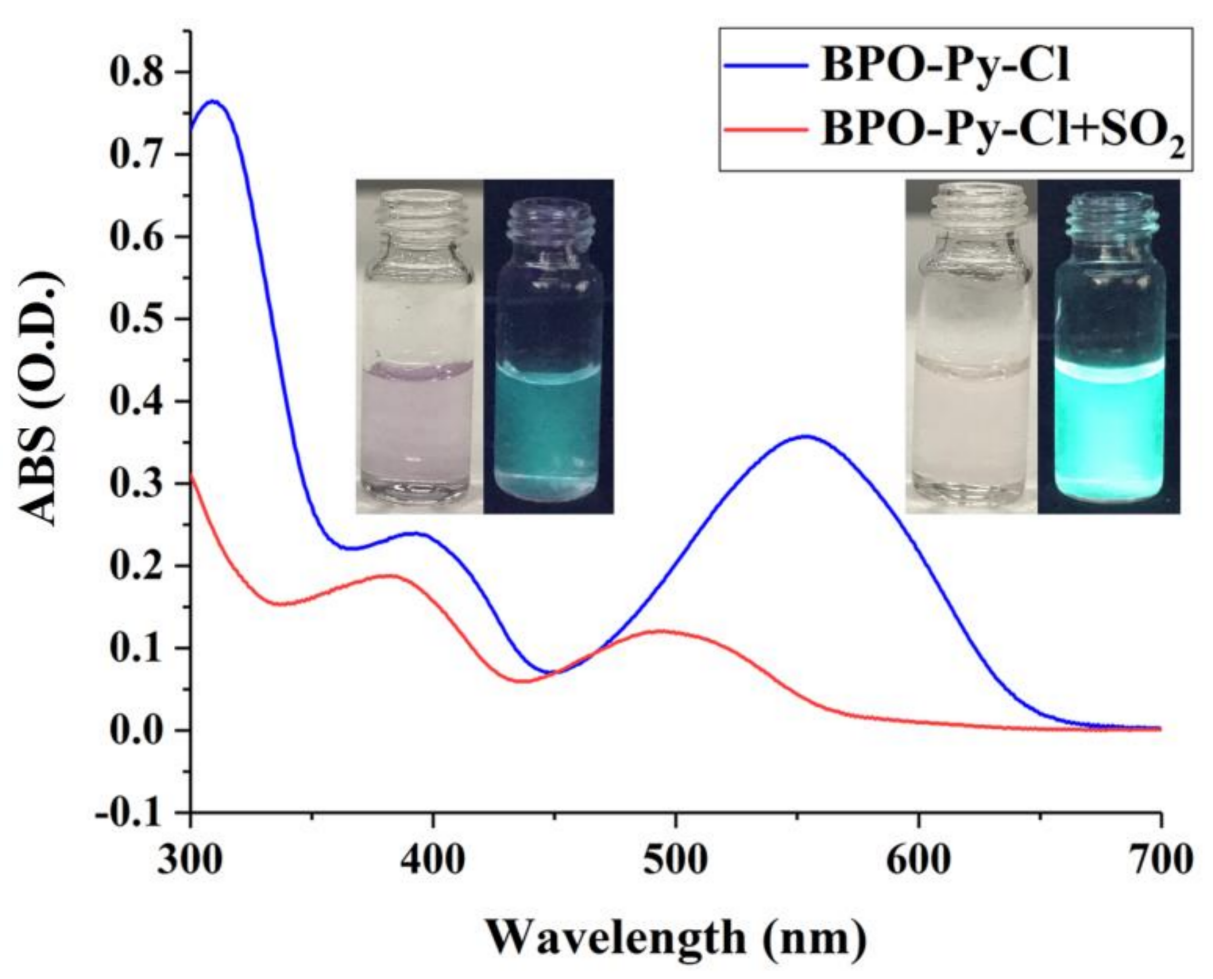
The absorption spectra and colorimetric changes of **BPO-Py-Cl** (10 μM) interacting with SO_2_ (500 μM) under visible and 365 nm ultraviolet lamps.

The optical property of **BPO-Py-Cl** (10 μM) was explored and the maxima absorption at 552 and 391 nm were observed in 10 mmol PBS (pH 7.4) buffer solution containing 20% DMSO as co-solvent (Figure 1). After interacting with 500 μM sulphite, the colour of the solution changed from purple to pale yellow under visible light and green fluorescence increased prominently under a 365 nm ultraviolet lamp. The absorption peaks of **BPO-Py-Cl** were blue-shifted to 493 nm and 379 nm, respectively. These results demonstrated that **BPO-Py-Cl** could detect SO_2_ efficiently.

### 2.3. pH-Dependent Fluorescence Response of the Probe toward SO_2_

**Figure 2:**
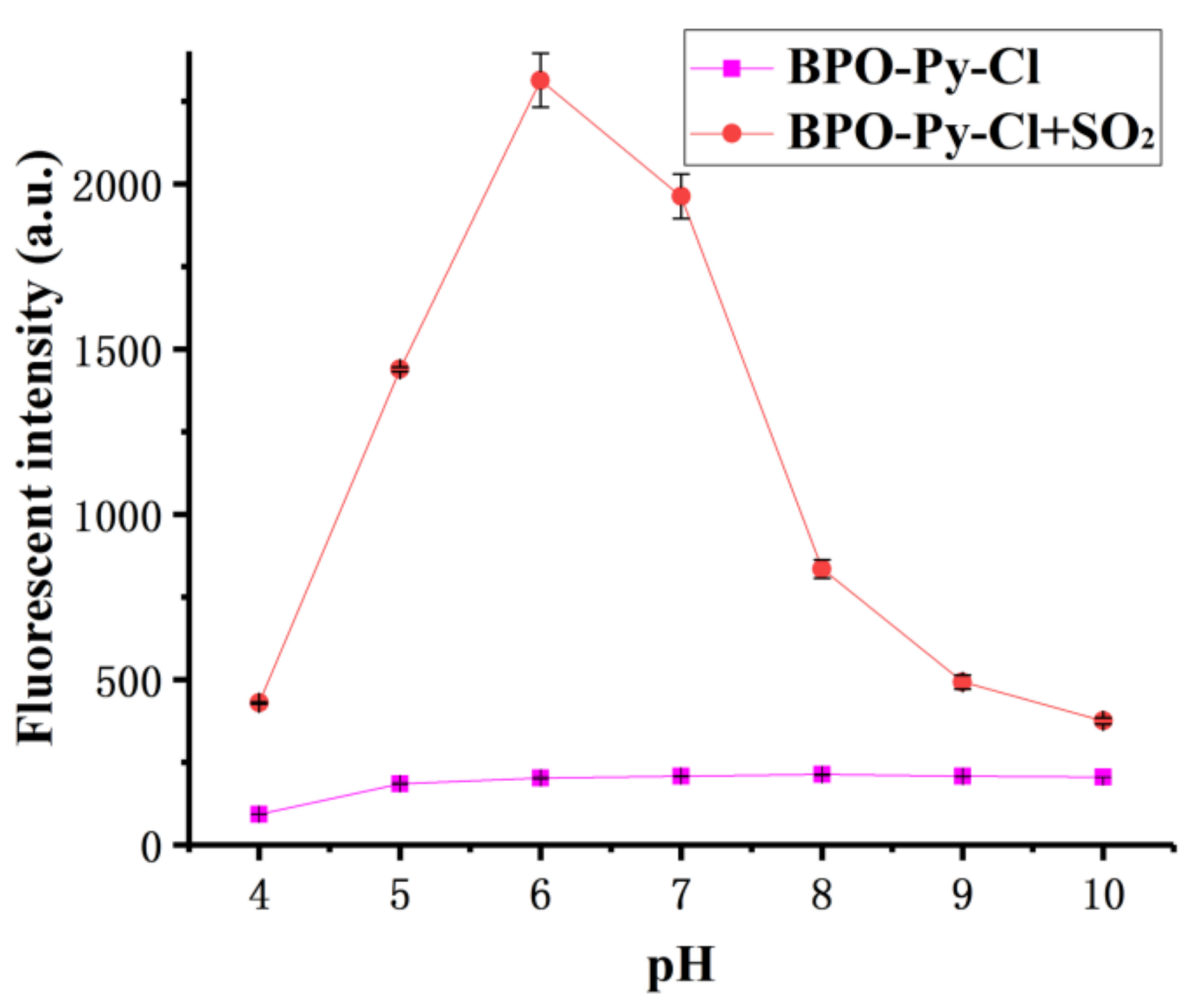
The pH dependent fluorescent changes of **BPO-Py-Cl** (10 μM) in PBS or after addition of SO_2_ (500 μM). The experiment was repeated three times (±S.D.).

The effect of pH on the fluorescent properties was examined. As shown in Figure 2, the fluorescent intensity of the **BPO-Py-Cl** had almost no remarkable changes in 10 mmol PBS buffer solution containing 20% DMSO when the pH increased from 4.0 to 10.0. However, upon addition of 500 μM Na_2_SO_3_, the fluorescent intensity at 495 nm exhibited conspicuous enhancement when pH increased from 4.0 to 6.0. When the pH continuously increased to 10.0, the fluorescent intensity decreased dramatically. Therefore, pH 6.0 was chosen as the optimal condition for **BPO-Py-Cl**.

### 2.4. Quantitative Determination of SO_2_

**Figure 3:**
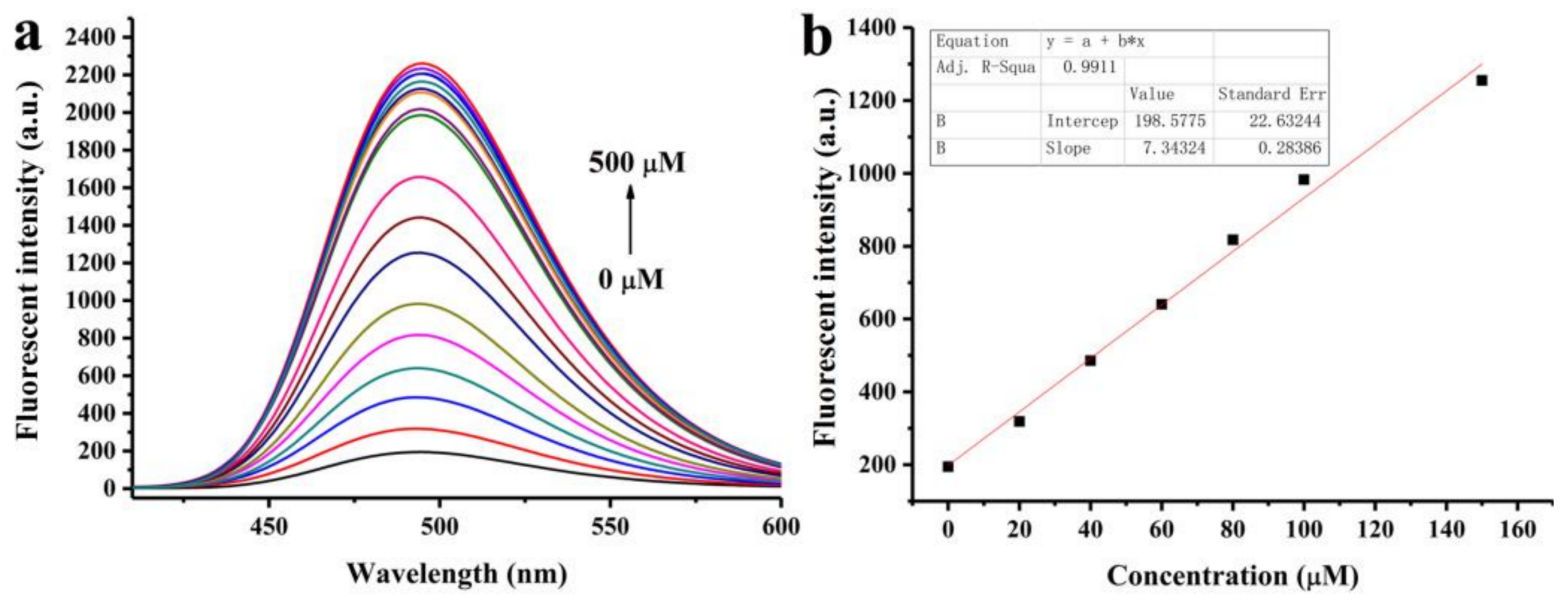
(**a**) Fluorescence titration of **BPO-Py-Cl** (10 µM) with SO_3_^2−^ (0, 20, 40, 60, 80, 100, 150, 200, 250, 300, 350, 400, 450, 500 µM) (λ_ex_ = 390 nm, slit: 2.5 nm/5 nm) in a 10 mM PBS:DMSO = 8:2 pH 6.0 buffer solution; and (**b**) linear fit of fluorescence intensity changes with Na_2_SO_3_. λ_ex_ = 390 nm. Slit: 2.5 nm/5 nm.

The fluorescent titrations were conducted in 10 mmol pH 6.0 PBS buffer containing 20% DMSO as a co-solution. As expected, the fluorescence intensity of **BPO-Py-Cl** at 495 nm increased gradually along with the increment of Na_2_SO_3_. To our delight, 400 μM Na_2_SO_3_ can lead to an intensity increase as much as 11.7 times greater (Figure 3a and Figure S1). The quantum yields increased from 0.043 to 0.673 after interacting with 400 μM Na_2_SO_3_. In the given concentration rage from 0 to 160.0 μM, the fluorescent signal intensity was linearly related to the concentration of Na_2_SO_3_ (Figure 3b), with the detection limit of 99.27 nM.

### 2.5. Selectivity Experiments

**Figure 4:**
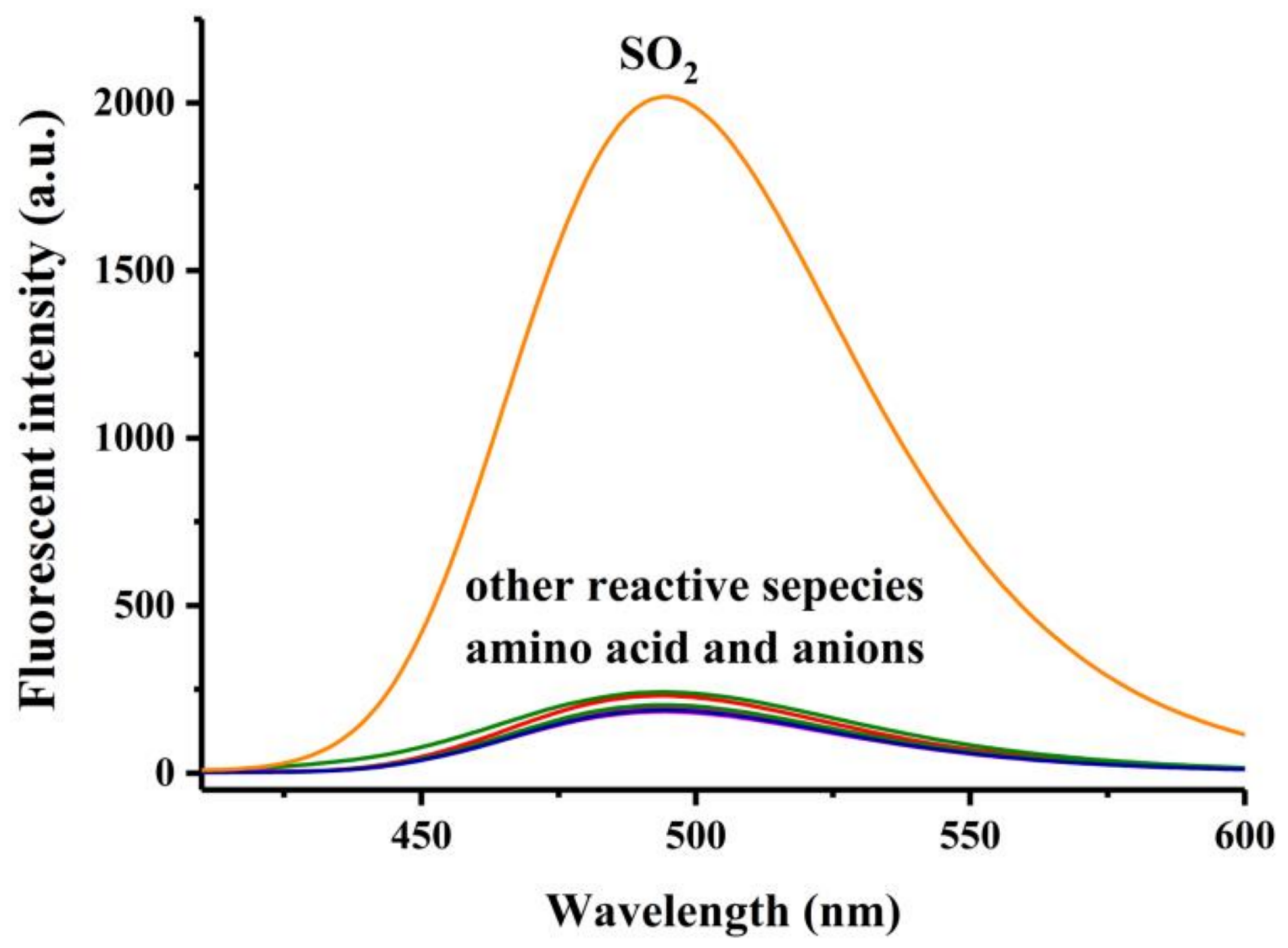
Fluorescence spectrum changes of **BPO-Py-Cl** (10 μM) interacting with 500 μM amino acids (Arg, Met, Ser, Asp, Gly, Ala, His, Val, Lys, Leu, Glu, Pro, Ile, Phe), reactive oxygen species (H_2_O_2_, NaClO, TBHP), reactive nitrogen species (NO_3_^−^, NO_2_^−^), and reactive sulphur species (SO_4_^2−^, Cys, Hcy, GSH, H_2_S, SO_2_) in 10 mM pH 6.0 PBS buffer solution containing 20% DMSO, λ_ex_ = 390 nm. Slit: 2.5 nm/5 nm.

**Figure 5:**
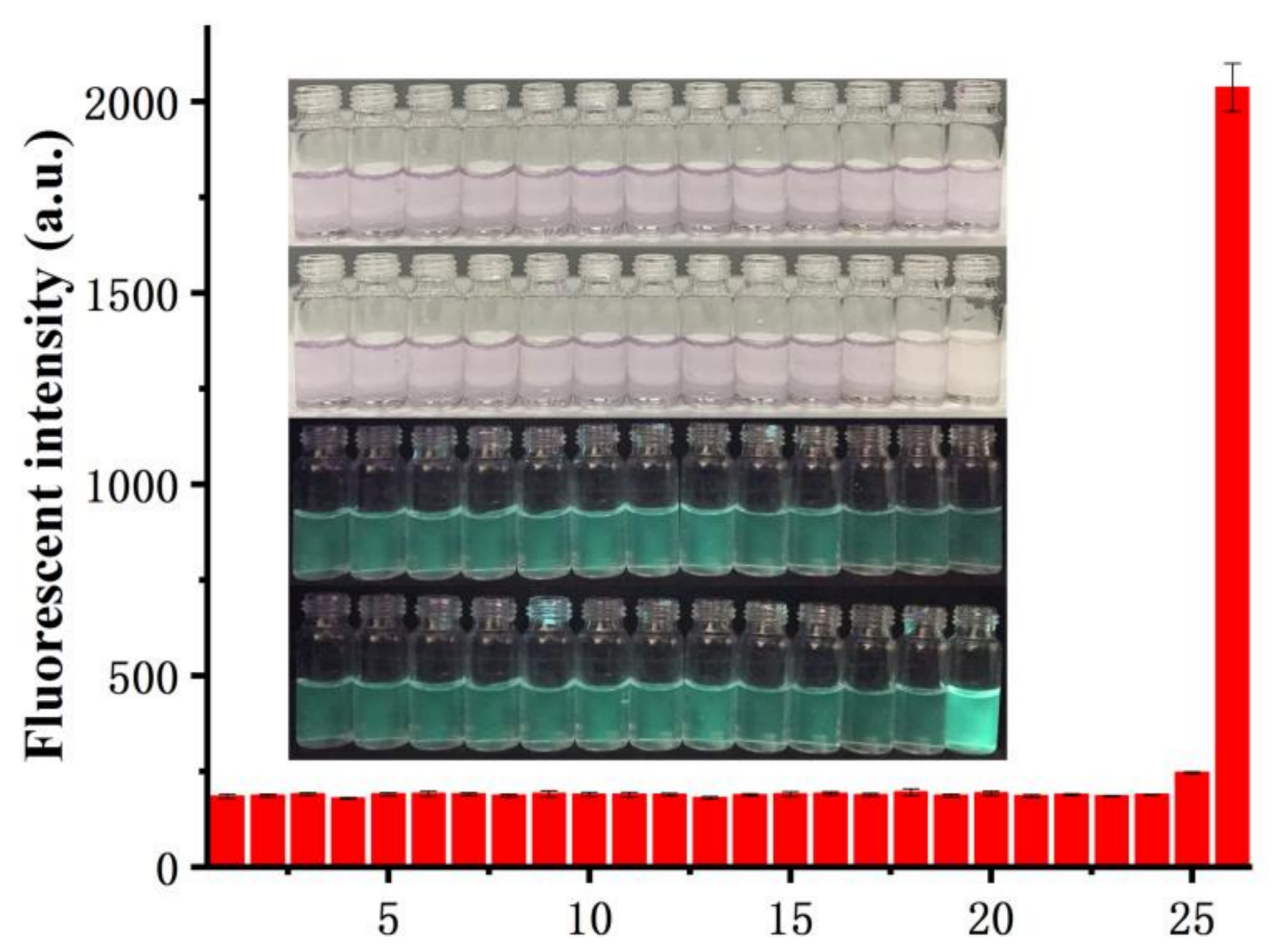
Fluorescence intensity changes of **BPO-Py-Cl** with SO_2_ and other species. Inset: fluorescence changes of **BPO-Py-Cl** upon addition of 500 μM SO_2_ and other species under visible light and UV light (Ex = 365 nm). 1. **BPO-Py-Cl**, 2. Ala, 3. Arg. 4. Asp, 5. Glu, 6. Gly, 7. His, 8. Ile, 9. Leu, 10. Lys, 11. Met, 12. Phe, 13. Pro, 14, Ser, 15. Val, 16. H_2_O_2_, 17, NaClO, 18. TBHP, 19. NO_3_^−^, 20. NO_2_^−^, 21. SO_4_^2−^, 22. Cys, 23. Hcy, 24. GSH, 25. H_2_S, 26. SO_2_. The experiment was repeated three times (±S.D.).

Based on the excellent fluorescent properties observed with Na_2_SO_3_, the selectivity of the probe for SO_2_ over other reactive species and amino acids were examined. As shown in Figure 4 and Figure S2, 500 μM sulphite could lead to noteworthy fluorescent spectrum changes of **BPO-Py-Cl**. Meanwhile, 500 μM of representative anions (F^−^, Cl^−^, Br^−^, I^−^, CO_3_^2−^, AcO^−^, PO_4_^3−^, SCN^−^, S_2_O_3_^2−^), biologically-abundant metal ions (Li^+^, Na^+^, K^+^, Mg^2+^, Ca^2+^, Cu^2+^, Zn^2+^, Ni^2+^, Fe^2+^, Fe^3+^, Pb^2+^), reactive oxygen species (NaClO, H_2_O_2_, TBHP), reactive nitrogen species (NO_2_^−^ and NO_3_^−^), other reactive sulphur species (SO_4_^2−^, H_2_S, Cys, HCy, and GSH) and amino acids (Ala, Arg, Asp, Glu, Gly, His, Ile, Leu, Lys, Met, Phe, Pro, Ser, Val) hardly led to any change. Figure 5 shows the fluorescent intensity and colour changes of the **BPO-Py-Cl** before and after the addition of sulphite or other species under visible light and 365 nm UV lamp. Obviously, only sulphite caused a remarkable colour change under the UV lamp.

### 2.6. Possible Mechanism

**Figure 6:**
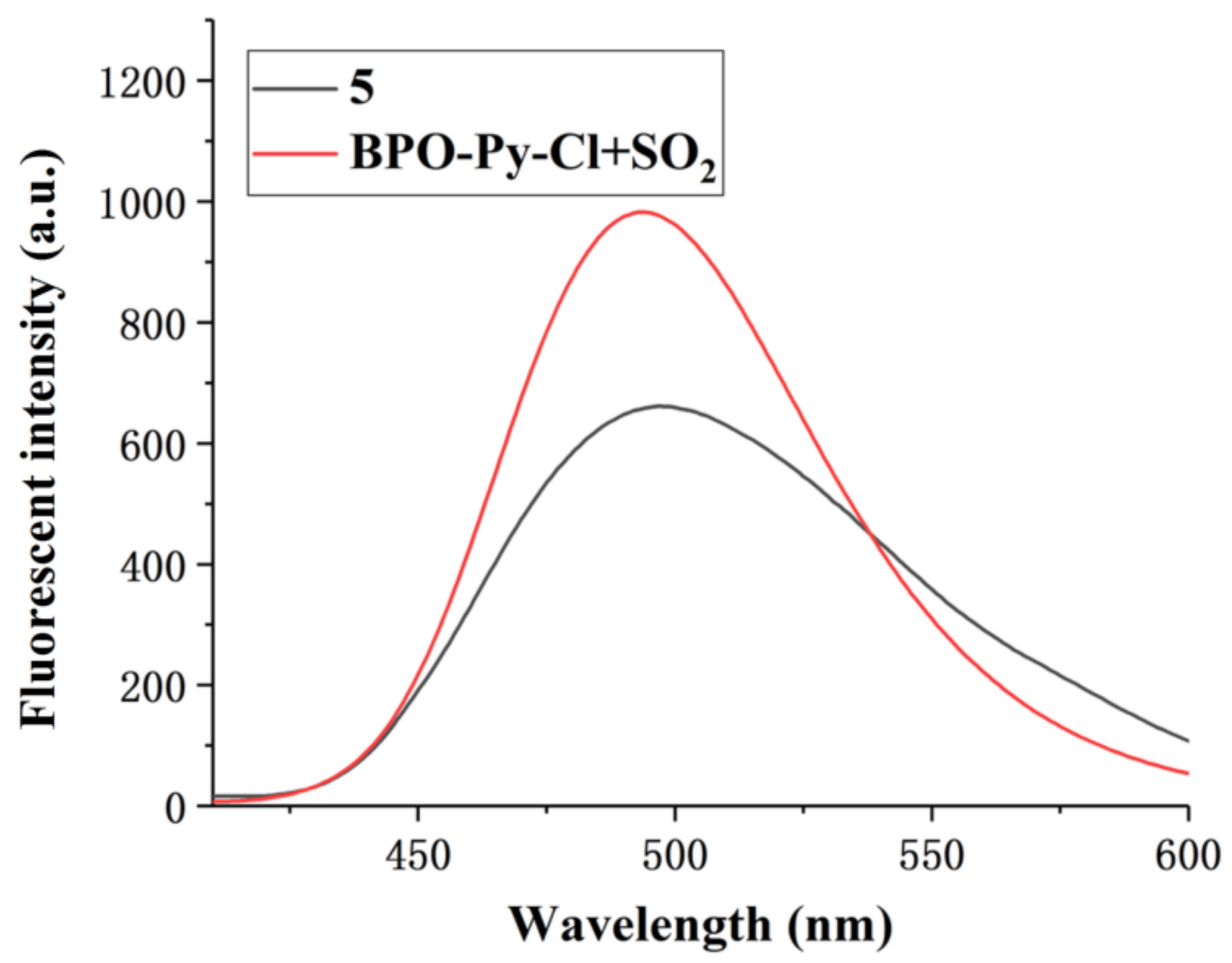
The fluorescent spectrum of compound **5** and **BPO-Py-Cl** interacting with SO_2_.

As reported by Lin, the carbon–carbon double bond linked with oxonium can provide the latent nucleophilic addition site for SO_2_ [37]. From the absorption spectrum (Figure 1), it can be speculated that Na_2_SO_3_ broke the conjugate system of **BPO-Py-Cl**. To verify the nucleophilic addition reaction of sulphite to the carbon–carbon double bond, the spectrum of compound **5** was investigated in pH 6.0 PBS buffer solution containing 20% DMSO. As we can see in Figure 6 and Figure S3, compared with the **BPO-Py-Cl** interacting with SO_2_, compound **5** had a similar excitation and emission wavelength, which demonstrated that both of them have similar conjugated systems. Therefore, nucleophilic addition reactions of sulphite to the carbon–carbon double bond was considered as the possible mechanism.

### 2.7. Living Cell Imaging

**Figure 7:**
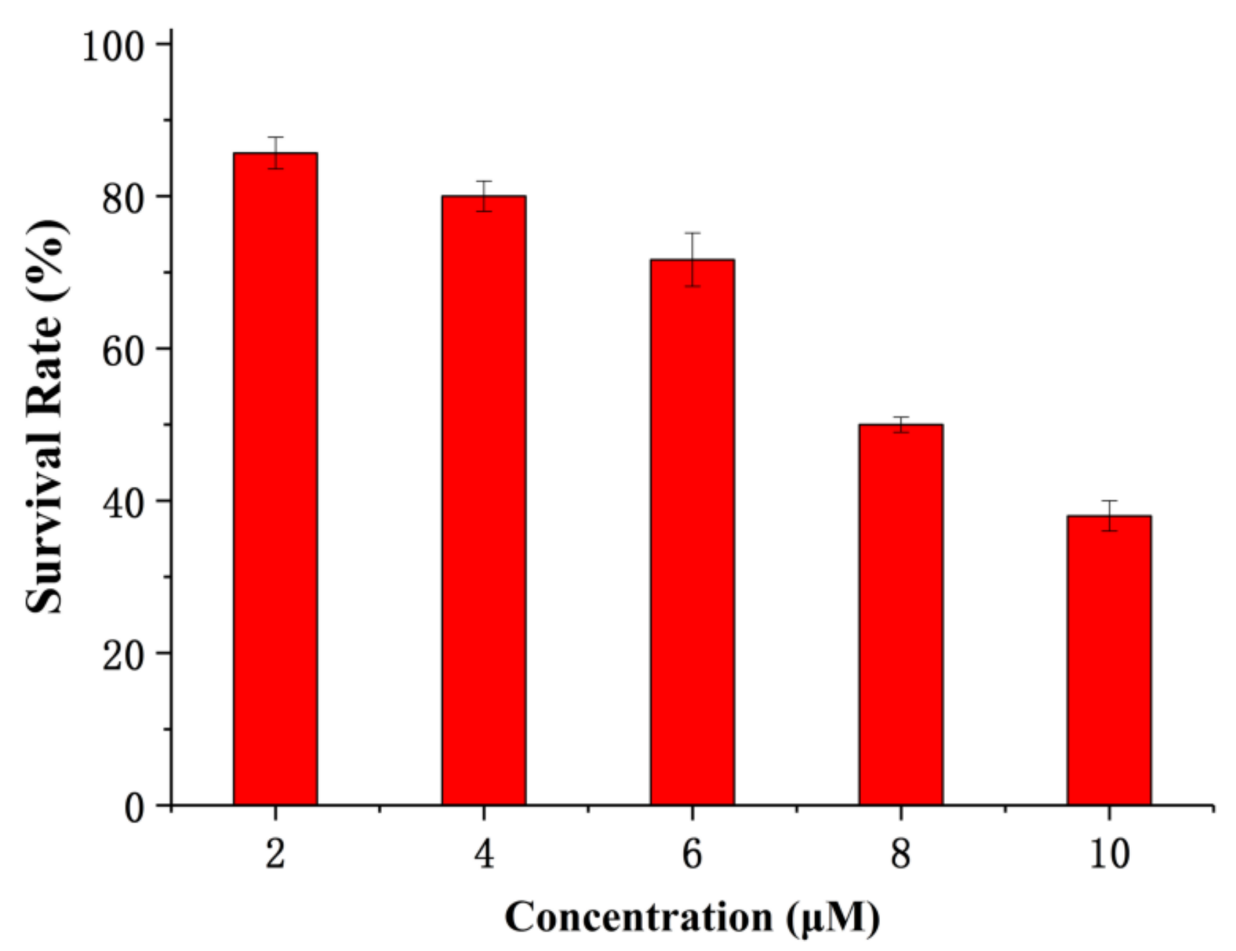
Cell viability by a standard CCK-8 assay. The experiment was repeated three times (±S.D.).

Encouraged by the aforementioned results, we further performed the capacity of probe for the fluorescent imaging in living cells. First, a standard CCK-8 assay in HeLa cells was investigated at different concentrations of **BPO-Py-Cl**. Figure 7 shows the changes of the cell survival rate with the concentration of **BPO-Py-Cl** increasing, and the cell survival rate was about 85% after incubation with 2 μM **BPO-Py-Cl** for 24 h. With 10 μM **BPO-Py-Cl**, it decreased to 40%.

**Figure 8:**
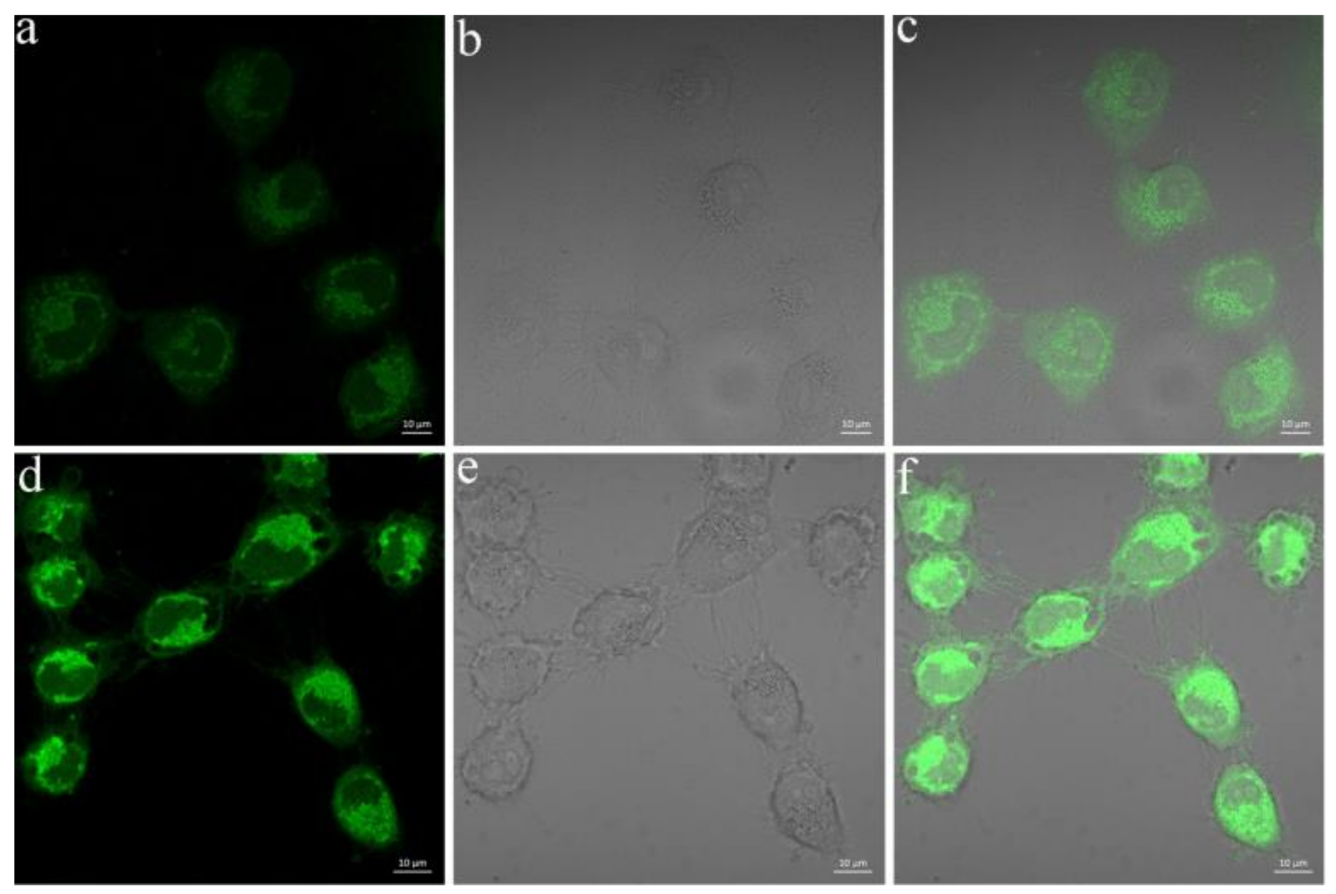
Cell image experiments of **BPO-Py-Cl**. HeLa cells were incubated with 10 μM **BPO-Py-Cl** for 30 min, (**a**–**c**) green channel, bright field, and the merged channel of green and bright field channels. HeLa cells were incubated with 10 μM **BPO-Py-Cl** for 30 min, then washed with PBS and incubated with SO_3_^2−^ (500 μM) for another 90 min; (**d**–**f**) are the green channel, bright field, and the merged channel of green and bright field channels.

Finally, the utility of **BPO-Py-Cl** to image SO_2_ was next carried out for living cells (Figure 8). The HeLa cells were incubated with **BPO-Py-Cl** (10 μM) in culture medium for 30 min at 37 °C, and exhibited weak fluorescence in the green channel. In a control experiment, the cells were pre-treated with **BPO-Py-Cl** for 30 min and further incubated with Na_2_SO_3_ (500 μM) for another 90 min. Obvious fluorescence increases in the green channel were observed compared with **BPO-Py-Cl** alone. These results demonstrated that **BPO-Py-Cl** could be used for the detection and imaging of SO_2_ in living cells.

## 3. Materials and Methods

### 3.1. Materials and General Instruments

All chemicals and reagents were purchased from commercial suppliers Aladdin-Reagent (Shanghai, China), Sigma-Aldrich (St. Louis, MO, USA), and TCI (Shanghai, China) and used without any further purification if not declared. All the solvents for optical spectroscopic studies were either HPLC or spectroscopic grade. TLC analyses were performed on GF 254silica gel. Silica gel (HG/T2354-92, 200–300 mesh) used for column chromatographic purifications were obtained from Qingdao Haiyang chemical Co., Ltd (Shandong, China). High-performance mass spectra were performed on a Bruker Daltonics Bio TOF (Karlsruhe, Germany) mass spectrometer in electrospray ionization mode. NMR spectra were recorded on a Bruker AMX-400 instrument using tetramethyl silane as the internal reference for ^1^H-NMR (400 MHz) or CDCl_3_ as the internal standard for ^13^C-NMR (100 MHz). UV–VIS absorption spectra and fluorescent spectra were recorded by a UV-1900 UV–VIS spectrophotometer and F4600 spectrofluorimeter with a 10 mm quartz cuvette from Hitachi PharmaSpec (Tokyo, Japan).

### 3.2. Synthesis and Characterization

*2-(4-(Diethylamino)-2-hydroxybenzoyl)benzoic acid* (**3**): 16.5 g (100 mmol) 3-diethylamino phenol, 19.0 g (128 mmol) phthalic anhydride, and 70 mL toluene were added to a 250-mL round round-bottom flask which was heated to 80 °C for 10 h, 90 °C for 5 h, 100 °C for 2 h, and 110 °C for 1 h, successively under N_2_. After cooling to RT, the precipitate was filtered off and washed with PhMe to obtain 28 g crude purple solid (compound **3**) (89.5% yield) which was used for the next reaction without purification.

*9-(2-Carboxyphenyl)-6-(diethylamino)-1,2,3,4-tetrahydroxanthylium perchlorate* (**4**): concentrated H_2_SO_4_ (70 mL) was cooled to 0 °C. Then, 6.6 mL of cyclohexanone (63.7 mmol) and 9.5 g of compound **3** (32 mmol) were added dropwise to the cooled H_2_SO_4_, respectively, with vigorous stirring. The mixture was further stirred at 90 °C for 1.5 h, cooled down to room temperature, and poured onto ice (300 g). The resulting precipitate was filtered off and washed with cold water (100 mL) after 7 mL 70% perchloric acid was added to the ice mixture. The precipitate was further purified by silica gel column chromatography with DCM to DCM/MeOH (10/1, *v*/*v*). About 9.4 g of purple solid (compound **4**) was obtained (62% yield).

*(E)-9-(2-Carboxyphenyl)-6-(diethylamino)-4-(4-hydroxybenzylidene)-1,2,3,4-tetrahydroxanthylium perchlorate* (**BPOH**): 4-hydroxybenzaldehyde (0.53 g, 4.32 mmol) and 1.72 g (3.60 mmol) of compound **5** were mixed in 40 mL AcOH. Then the mixture was heated to 90 °C overnight under N_2_. Subsequently, the solvent was removed under reduced pressure. After that, the crude product was dissolved in 50 mL dichloromethane, washed with water (50 mL) three times, and dried by sodium sulphate. The crude product was concentrated and purified with chromatography on a silica gel column with DCM to DCM/MeOH (10/1, *v*/*v*) to get 1.35 g **BPOH** as dark purple solid (65% yield).

*(E)-9-(2-Carboxyphenyl)-4-(4-((4-chloropyridin-2-yl)oxy)benzylidene)-6-(diethylamino)-1,2,3,4-tetrahydroxanthylium* (**BPO-Py-Cl**): About 174.0 mg (0.3 mmol) of BPOH was dissolved in 10 mL of DMF. Then, 441.0 mg (3.0 mmol) of 2,4-dichloropyridine and 207 mg (1.5 mmol) of K_2_CO_3_ were added into the above solution. After reacting for 3–5 h at 50 °C under supervision by TLC, the solvent was removed under reduced pressure to obtain the crude product which as purified on a silica gel column chromatography with DCM to DCM/MeOH (20/1, *v*/*v*) to obtain an aubergine solid of 70 mg **BPO-Py-Cl** (33.3% yield). ^1^H-NMR (400 MHz, CDCl_3_), 8.28 (d, 1H, *J* = 5.6 Hz), 7.99 (d, 1H, *J* = 7.6 Hz), 7.68 (t, 1H, *J* = 7.6 Hz), 7.58 (t, 1H, *J* = 7.2 Hz), 7.49 (d, 2H, *J* = 8.4 Hz), 7.41 (s, 1H), 7.27 (d, 1H, *J* = 7.6 Hz), 7.13 (d, 2H, *J* = 8.4 Hz), 6.89–6.85 (m, 2H), 6.53 (d, 1H, *J* = 8.8 Hz), 6.45 (d, 1H, *J* = 2.4 Hz), 6.39 (dd, 1H, *J* = 2.8 Hz, *J* = 8.8 Hz), 3.39 (q, 4H, *J* = 6.8 Hz), 2.86–2.82 (m, 1H), 2.69–2.65 (m, 1H), 2.11–2.07 (m, 1H), 1.75–1.63 (m, 3H), 1.20 (t, 4H, *J* = 6.8 Hz). ^13^C-NMR (100 MHz, CDCl_3_), 170.1, 166.4, 152.8,152.3, 152.2, 150.8, 149.4, 146.8, 134.5, 131.5, 131.2, 129.3, 128.6, 123.5, 120.6, 112.0, 111.5, 108.9, 108.5, 104.8, 97.3, 44.5, 27.2, 23.1, 22.4, 12.6. HRMS (ESI): *m*/*z* [M]^+^ calcd. for C_36_H_32_ClN_2_O_4_: 591.2045; found 591.2043.

### 3.3. Preparation of the Test Solutions

Na_2_SO_3_ was used as the source of SO_2_. A probe stock solution of the **BPO-Py-Cl** (2.0 mM) was prepared in DMSO. Other stock solutions of representative anions (F^−^, Cl^−^, Br^−^, I^−^, CO_3_^2−^, AcO^−^, PO_4_^3−^, SCN^−^, S_2_O_3_^2−^), biologically-abundant metal ions (Li^+^, Na^+^, K^+^, Mg^2+^, Ca^2+^, Cu^2+^, Zn^2+^, Ni^2+^, Fe^2+^, Fe^3+^, Pb^2+^), reactive oxygen species (NaClO, H_2_O_2_, TBHP), reactive nitrogen species (NO_2_^−^ and NO_3_^−^), other reactive sulphur species (SO_4_^2−^, H_2_S, Cys, HCy, and GSH), and amino acids (Lys, Glu, Leu, Met, Arg, His, Phe, Ser, Ile, Val, Ala, Asp, Pro,) were prepared in deionized water at a concentration of 50 mM. All the test solutions were prepared by mixing 75 μL of each species stock solution and 15 μL of the probe stock solution in 3.0 mL with PBS buffer/DMSO (8:2, *v*/*v*) solution. The absorption and emission spectra were recorded after the test solution was incubated at room temperature for 2 h.

### 3.4. Quantum Yields

The fluorescence quantum yield (Φ) was calculated using the following formula:

Φ_x_ = Φ_s_ × (A_s_/A_x_) × (D_x_/D_s_) × (n_x_^2^/n_s_^2^)

where the subscripts s and x refer to the standard material and test sample, respectively. Φ_s_ and Φ_x_ represent the fluorescence quantum yield of the standard and test sample; ns and nx represent the refractive index of the solvents used; A_s_ and A_x_ represent the absorption intensity at the excitation wavelength of the standard and test sample; D_s_ and D_x_ represent the integral of the fluorescence intensity of the standard and test sample. Fluorescein in 0.1 M NaOH (Φ_s_ = 0.85) was used as the standard [43].

### 3.5. CCK-8 Assay for the Cell Cytotoxicity

The cytotoxicity of the probe was determined by CCK-8 assays. HeLa cells were first seeded in 96-well plates (about 7000 cells per well) and cultured overnight for 70–80% cell confluence. After the cells were incubated with **BPO-Py-Cl** at different concentrations (2, 4, 6, 8, 10 μM in DMSO/cell culture medium = 1:49) for twenty-four hours, 10 μL of CCK-8 mixed in 90 μL of PBS was added to each well of the 96-well assay plate for additional 1 h incubation at 37 °C. The absorbance was measured at a wavelength of 450 nm using an ELISA plate reader (Model 550, BioRad, Hercules, CA, USA). All the samples were repeated three times.

### 3.6. Determination of the Detection Limit

The detection limit was calculated based on the fluorescence titration [44] with the following equation:

detection limit = 3σ_bi_/m

where σ_bi_ is the standard deviation of blank measurements, m is the slope between intensity versus sample concentration (signal-to-noise ratio of 3:1). The standard deviation of blank measurements was determined when the emission intensity of probe (10 μM) without Na_2_SO_3_ was measured 10 times. Then, fluorescent titrations were conducted under the present conditions. A good linear relationship between the fluorescence intensity and the concentration of Na_2_SO_3_ was obtained, the slope between intensity versus Na_2_SO_3_ concentration was m in the above-mentioned formula.

### 3.7. Fluorescence Imaging of SO_2_ in Living Cells

HeLa cells were incubated in DMEM medium (which contained 10% foetal bovine serum and 1% antibiotic-antimycotic) at 37 °C in an incubator (5% CO_2_/95% air). Then cells were seeded in confocal dishes (4 × 10^3^ per well) and incubated for 24 h. Before staining, the cells were washed three times with physiological PBS and incubated with **BPO-Py-Cl** (10 μM) for 30 min at 37 °C. After being washed with physiological PBS three times to remove free probes, cell imaging was carried out after the cells were further incubated with Na_2_SO_3_ (500 μM) for another 90 min. Meanwhile, the control experiment was performed. The cells were incubated with **BPO-Py-Cl** (10 μM) for 30 min and then washed with PBS three times. Then, cell imaging was performed. The confocal fluorescent images were recorded for the green channel, corresponding to the wavelength range 460–530 nm, which was monitored for excitation at 405 nm.

## 4. Conclusions

In summary, a novel chromenylium derivative bearing the electron-withdrawing group **BPO-Py-Cl** was synthesized, and the probe exhibited a remarkable turn-on fluorescence response toward SO_2_ with a 105 nm Stock shift. It exhibited high sensitivity and selectivity with a low detection limit (99.27 nM) for SO_2_. In addition, the probe was cell membrane permeable and could be successfully applied to the imaging of SO_2_ in living cells.

## Supplementary Materials

The supplementary materials are available online.

## References

[1] Chen T.-M., Kuschner W.G., Gokhale J., Shofer S. (2007). Outdoor air pollution: Nitrogen dioxide, sulfur dioxide, and carbon monoxide health effects. Am. J. Med. Sci..

[2] Li X., Bazer F.W., Gao H., Jobgen W., Johnson G.A., Li P., McKnight J.R., Satterfield M.C., Spencer T.E., Wu G. (2009). Amino acids and gaseous signalling. Amino Acids.

[3] Griffith O.W. (1987). Mammalian sulfur amino acid metabolism: An overview. Methods Enzymol..

[4] Li J., Meng Z. (2009). The role of sulfur dioxide as an endogenous gaseous vasoactive factor in synergy with nitric oxide. Nitric Oxide.

[5] Li J., Li R., Meng Z. (2010). Sulfur dioxide upregulates the aortic nitric oxide pathway in rats. Eur. J. Pharmacol..

[6] Song A., Liao Q., Li J., Lin F., Liu E., Jiang X., Deng L. (2012). Chronic exposure to sulfur dioxide enhances airway hyperresponsiveness only in ovalbumin-sensitized rats. Toxicol. Lett..

[7] Jin H., Wang Y., Wang X., Sun Y., Tang C., Du J. (2013). Sulfur dioxide preconditioning increases antioxidative capacity in rat with myocardial ischemia reperfusion (I/R) injury. Nitric Oxide.

[8] Li W., Tang C., Jin H., Du J. (2011). Regulatory effects of sulfur dioxide on the development of atherosclerotic lesions and vascular hydrogen sulfide in atherosclerotic rats. Atherosclerosis.

[9] Sun Y., Tian Y., Prabha M., Liu D., Chen S., Zhang R., Liu X., Tang C., Tang X., Jin H. (2010). Effects of sulfur dioxide on hypoxic pulmonary vascular structural remodelling. Lab. Investig..

[10] Liang Y., Liu D., Ochs T., Tang C., Chen S., Zhang S., Geng B., Jin H., Du J. (2011). Endogenous sulfur dioxide protects against isoproterenol-induced myocardial injury and increases myocardial antioxidant capacity in rats. Lab. Investig..

[11] Li G.K., Sang N. (2009). Delayed rectifier potassium channels are involved in SO_2_ derivative-induced hippocampal neuronal injury. Ecotoxicol. Environ. Saf..

[12] Migliore L., Coppedè F. (2009). Environmental-induced oxidative stress in neurodegenerative disorders and aging. Mutat. Res..

[13] Sang N., Yun Y., Yao G., Li H., Guo L., Li G. (2011). SO_2_-induced neurotoxicity is mediated by cyclooxygenases-2-derived prostaglandin E_2_ and its downstream signaling pathway in rat hippocampal neurons. Toxicol. Sci..

[14] Liu D., Huang Y., Bu D., Liu A.D., Holmberg L., Jia Y., Tang C., Du J., Jin H. (2014). Sulfur dioxide inhibits vascular smooth muscle cell proliferation via suppressing the Erk/MAP kinase pathway mediated by cAMP/PKA signaling. Cell Death Dis..

[15] Yu F., Han X., Chen L. (2014). Fluorescent probes for hydrogen sulfide detection and bioimaging. Chem. Commun..

[16] Lin V.S., Chen W., Xian M., Chang C.J. (2015). Chemical probes for molecular imaging and detection of hydrogen sulfide and reactive sulfur species in biological systems. Chem. Soc. Rev..

[17] Jiao X., Li Y., Niu J., Xie X., Wang X., Tang B. (2018). Small-molecule fluorescent probes for imaging and detection of reactive oxygen, nitrogen, and sulfur species in biological systems. Anal. Chem..

[18] Sun W., Guo S., Hu C., Fan J., Peng X. (2016). Recent development of chemosensors based on cyanine platforms. Chem. Rev..

[19] Hou J.-T., Ren W.X., Li K., Seo J., Sharma A., Yu X.-Q., Kim J.S. (2017). Fluorescent bioimaging of pH: From design to applications. Chem. Soc. Rev..

[20] Xu W., Zeng Z., Jiang J.-H., Chang Y.-T., Yuan L. (2016). Discerning the chemistry in individual organelles with small-molecule fluorescent probes. Angew. Chem. Int. Ed..

[21] Wu M.-Y., He T., Li K., Wu M.-B., Huang Z., Yu X.-Q. (2013). A real-time colorimetric and ratiometric fluorescent probe for sulfite. Analyst.

[22] Wu M.-Y., Li K., Li C.-Y., Hou J.-T., Yu X.-Q. (2014). A water-soluble near-infrared probe for colorimetric and ratiometric sensing of SO_2_ derivatives in living cells. Chem. Commun..

[23] Liu Y., Li K., Xie K.-X., Li L.-L., Yu K.-K., Wang X., Yu X.-Q. (2016). A water-soluble and fast-response mitochondria-targeted fluorescent probe for colorimetric and ratiometric sensing of endogenously generated SO_2_ derivatives in living cells. Chem. Commun..

[24] Liu Y., Li K., Wu M.-Y., Liu Y.-H., Xie Y.-M., Yu X.-Q. (2015). A mitochondria-targeted colorimetric and ratiometric fluorescent probe for biological SO_2_ derivatives in living cells. Chem. Commun..

[25] Yang J., Li K., Hou J.-T., Li L.-L., Lu C.-Y., Xie Y.-M., Wang X., Yu X.-Q. (2016). Novel tumor-specific and mitochondria-targeted near-infrared-emission fluorescent probe for SO_2_ derivatives in living cells. ACS Sens..

[26] Xu W., Teoh C.L., Peng J., Su D., Yuan L., Chang Y.-T. (2015). A mitochondria-targeted ratiometric fluorescent probe to monitor endogenously generated sulfur dioxide derivatives in living cells. Biomaterials.

[27] Sun Y.-Q., Liu J., Zhang J., Yang T., Guo W. (2013). Fluorescent probe for biological gas SO_2_ derivatives bisulfite and sulfite. Chem. Commun..

[28] Wu W.-L., Wang Z.-Y., Dai X., Miao J.-Y., Zhao B.-X. (2016). An effective colorimetric and ratiometric fluorescent probe based FRET with a large Stokes shift for bisulfite. Sci. Rep..

[29] Li D.-P., Wang Z.-Y., Cao X.-J., Cui J., Wang X., Cui H.-Z., Miao J.-Y., Zhao B.-X. (2016). A mitochondria-targeted fluorescent probe for ratiometric detection of endogenous sulfur dioxide derivatives in cancer cells. Chem. Commun..

[30] Li G., Chen Y., Wang J., Wu J., Gasser G., Ji L., Chao H. (2015). Direct imaging of biological sulfur dioxide derivatives in vivo using a two-photon phosphorescent probe. Biomaterials.

[31] Ma Y., Tang Y., Zhao Y., Gao S., Lin W. (2017). Two-photon and deep-red emission ratiometric fluorescent probe with a large emission shift and signal ratios for sulfur dioxide: Ultrafast response and applications in living cells, brain tissues, and zebrafishes. Anal. Chem..

[32] Zhang W., Liu T., Huo F., Ning P., Meng X., Yin C. (2017). Reversible ratiometric fluorescent probe for sensing bisulfate/H_2_O_2_ and Its application in zebrafish. Anal. Chem..

[33] Dou K., Fu Q., Chen G., Yu F., Liu Y., Cao Z., Li G., Zhao X., Xia L., Chen L. (2017). A novel dual-ratiometric-response fluorescent probe for SO_2_/ClO^−^ detection in cells and in vivo and its application in exploring the dichotomous role of SO_2_ under the ClO^−^ induced oxidative stress. Biomaterials.

[34] Zhu Y., Du W., Zhang M., Xu Y., Song L., Zhang Q., Tian X., Zhou H., Wu J., Tian Y. (2017). A series of water-soluble A–π–A′ typological indolium derivatives with two-photon properties for rapidly detecting HSO_3_^−^/SO_3_^2−^ in living cells. J. Mater. Chem. B.

[35] Che S., Dao R., Zhang W., Lv X., Li H., Wang C. (2017). Designing an anion-functionalized fluorescent ionic liquid as an efficient and reversible turn-off sensor for detecting SO_2_. Chem. Commun..

[36] Dou K., Chen G., Yu F., Sun Z., Li G., Zhao X., Chen L., You J. (2017). A two-photon ratiometric fluorescent probe for the synergistic detection of the mitochondrial SO_2_/HClO crosstalk in cells and in vivo. J. Mater. Chem. B.

[37] Shang H., Liu K., Lin W. (2017). Construction of a novel ratiometric near-infrared fluorescent probe for SO_2_ derivatives and its application for biological imaging. Anal. Methods.

[38] Chen H., Dong B., Tang Y., Lin W. (2017). A unique “integration” strategy for the rational design of optically tunable near-infrared fluorophores. Acc. Chem. Res..

[39] Dong B., Song X., Kong X., Wang C., Tang Y., Liu Y., Lin W. (2016). Simultaneous near-infrared and two-photon in vivo imaging of H_2_O_2_ using a ratiometric fluorescent probe based on the unique oxidative rearrangement of oxonium. Adv. Mater..

[40] Yuan L., Lin W., Yang Y., Chen H. (2012). A unique class of near-infrared functional fluorescent dyes with carboxylic-acid-modulated fluorescence ON/OFF switching: Rational design, synthesis, optical properties, theoretical calculations, and applications for fluorescence imaging in living animals. J. Am. Chem. Soc..

[41] Wei Y., Cheng D., Ren T., Li Y., Zeng Z., Yuan L. (2016). Design of NIR chromenylium-cyanine fluorophore library for “switch-ON” and ratiometric detection of bio-active species in vivo. Anal. Chem..

[42] Yuan L., Lin W., Chen H. (2013). Analogs of Changsha near-infrared dyes with large Stokes Shifts for bioimaging. Biomaterials.

[43] Parker B.C.A., Rees W.T. (1960). Correction of fluorescence spectra and measurement of fluorescence quantum efficiency. Analyst.

[44] Joshi B.P., Park J., Lee W.I., Lee K. (2009). Ratiometric and turn-on monitoring for heavy and transition metal ions in aqueous solution with a fluorescent peptide sensor. Talanta.

