# Supplementary material for: A Novel Colorimetric Fluorescent Probe for SO2 and Its Application in Living Cells Imaging
Source: Molecules. 2018 Apr 10;23(4):871. doi: 10.3390/molecules23040871 (PMC6017954; doi:10.3390/molecules23040871)

Supporting Information

A Novel Colorimetric Fluorescent Probe for SO_2 and Its Application in Living Cells Imaging

Ming-Yu Wu[1]*, Jing Wu[1], Yue Wang[1], Yan-Hong Liu[2], Xiao-Qi Yu[2]*

1. School of Life Science and Engineering, Southwest Jiaotong University, Chengdu 610064, China;

2. Key Laboratory of Green Chemistry and Technology, Ministry of Education, College of Chemistry, Sichuan University, Chengdu, 610064, China.

* Corresponding author. Ming-Yu Wu; Xiao-Qi Yu
* E-Mail: wumy1050hx@swjtu.edu.cn (M.-Y.W); xqyu@scu.edu.cn (X.-Q.Y.)

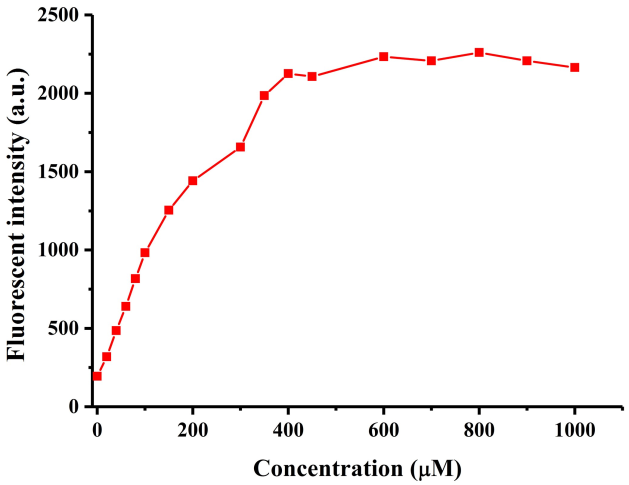

Figure S1 Fluorescence intensity changes for **BPO-Py-Cl** (10 μM) interacting with SO_3^{2-} (0, 20, 40, 60, 80, 100, 150, 200, 250, 300, 350, 400, 450, 500, 600, 700, 800, 900, 1000 μM) (λ_{ex} = 390 nm, λ_{em} = 495 nm, slit: 2.5 nm/5 nm) in 10 mM PBS:DMSO = 8:2 pH 6.0 buffer solution.

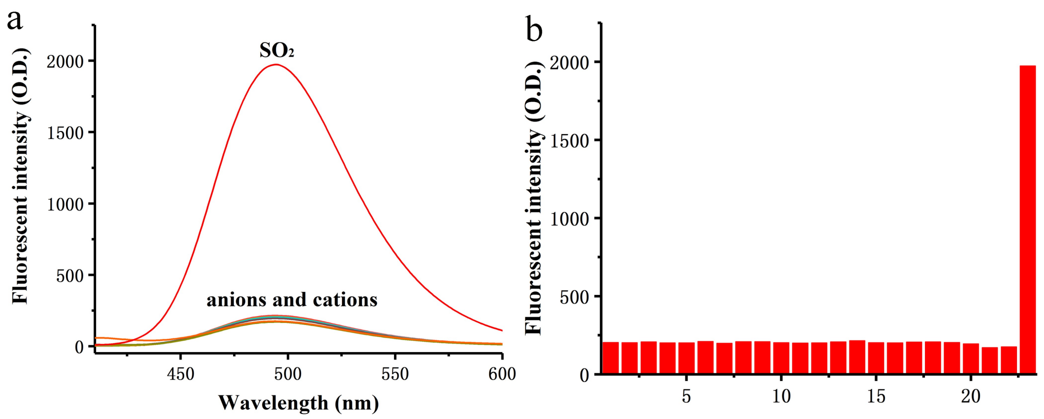

Figure S2. Fluorescence spectrum (**a**) and intensity (**b**) changes of **BPO-Py-Cl** with 500 μM SO_2, other representative anions and biologically abundant metal ions. 1. **BPO-Py-Cl**, 2. F^-, 3. Cl^-, 4. Br^-, 5. I^-, 6. CO_3^{2-}, 7.AcO^-, 8. PO_4^{3-}, 9.SCN^-, 10. $S_2O_3^{2-}$, 11. HS^-, 12. Li^+, 13. Na^+, 14. K^+, 15. Mg^{2+}, 16. Ca^{2+}, 17. Cu^{2+}, 18. Zn^{2+}, 19. Ni^{2+}, 20. Fe^{2+}, 21. Fe^{3+}, 22. Pb^{2+}, 23. SO_2.

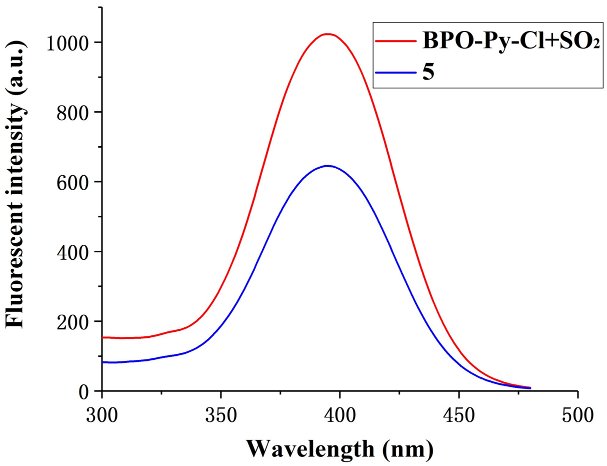

Figure S3. The excitation spectrum of compound **5** and **BPO-Py-Cl** interacting with SO_2.

^{1}H NMR, ^{13}C NMR and HRMS Spectra

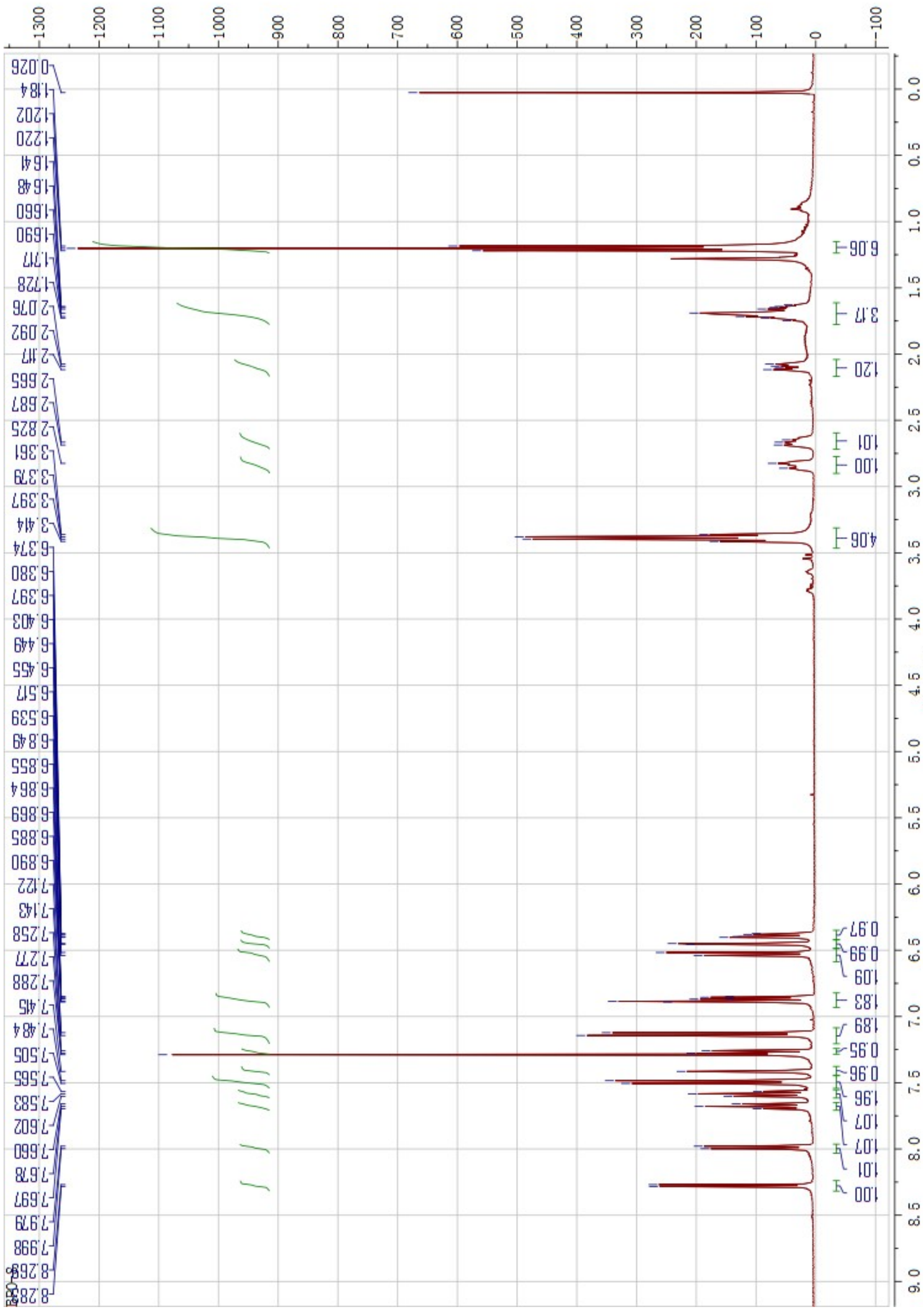

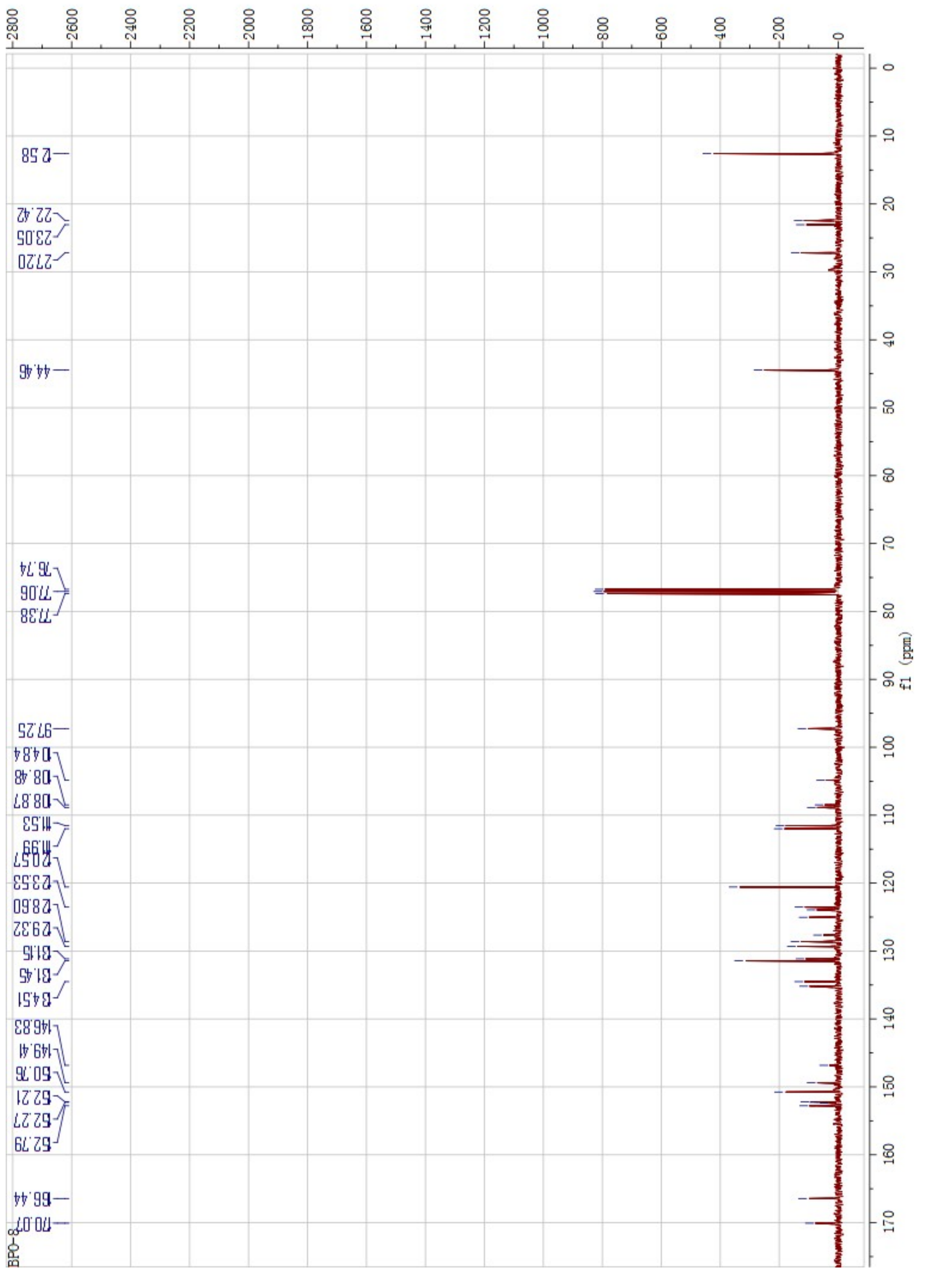
f1 (ppm)

MS(E+)

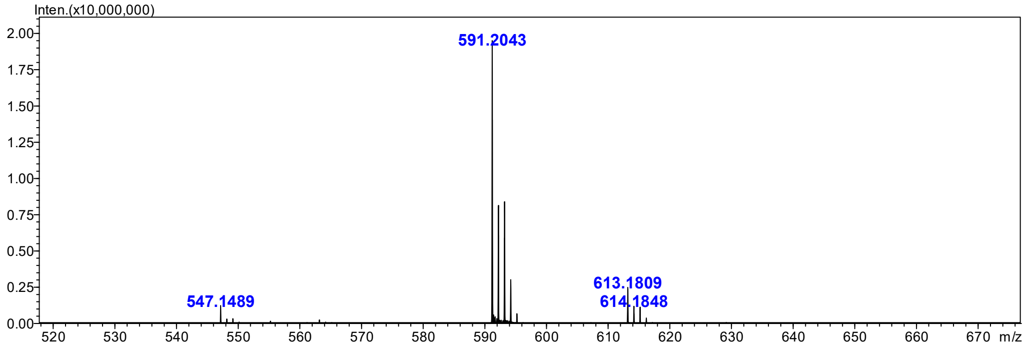

Supplement: Supplementary file 1 [file molecules-23-00871-s001.pdf]
